# Mental Imagery and Social Pain in Adolescents—Analysis of Imagery Characteristics and Perspective—A Pilot Study

**DOI:** 10.3390/children8121160

**Published:** 2021-12-08

**Authors:** Susan Muriel Schwarz, Mersiha Feike, Ulrich Stangier

**Affiliations:** Department of Clinical Psychology and Psychotherapy, Goethe University, Varrentrappstraße 40-42, 60486 Frankfurt am Main, Germany; mersi.feike@stud.uni-frankfurt.de (M.F.); stangier@psych.uni-frankfurt.de (U.S.)

**Keywords:** mental imagery, social pain, depression, anxiety, children, adolescents

## Abstract

Background: Mental imagery (MI) may play a key role in the development of various mental disorders in adolescents. Adolescence is known to be a fragile life period, in which acceptance by one’s favored peer group is extremely important, and social rejection is particularly painful. This is the first pilot study investigating MI and its relationship to social pain (SP). Method: A sample of 80 adolescents (14–20 years; 75.3% female) completed a web-based quasi-experimental design about the contents and characteristics of their spontaneous positive and negative MI and associated emotions, and were asked to complete the Social Pain Questionnaire, the Becks Depression Inventory and the Social Phobia Inventory. Results: A higher score of SP was significantly associated with increased fear, sadness, and feelings of guilt, and less control over negative MI. Characteristics of negative MI were more precisely predicted by SP scores than depression- and social anxiety scores. Adolescents with higher SP-scores more often reported negative images including social situations and were more likely to perceive negative images in a combination of field-and observer perspectives than adolescents with lower SP scores. Conclusion: SP-sensitivity seems to be linked to unique characteristics of negative MI, which reveals the strong emotional impact of social exclusion in youths. The results do not allow causal conclusions to be drawn, but raise questions about previous studies comparing each imagery perspective individually.

## 1. Introduction

Adolescence is known to be a fragile life period in which acceptance by one’s preferred peer group is more important than in other phases, and therefore, social rejection is especially painful [1,2]. The experience of pain as a consequence of interpersonal exclusion or ostracism, such as rejection by a peer group and bullying, or the loss of a loved one, is defined as social pain [3]. Social pain is one of the social emotions and can be triggered by actual or imagined situations with other people. Social emotions are emotions which a person has towards another person and they help acting communally and caring. Among these social emotions, a distinction is made between social-evaluative and social-relational emotions (in addition to positive and negative emotions). Social-evaluative emotions are sensations that a person feels towards other persons. These include, for example, hate or love. Social pain, like sadness, guilt, or shame, belongs to the social-relational emotions. These result from the perceived emotions of other persons toward oneself [4].

Social rejection by one’s preferred peer group, for instance, has negative consequences for our wellbeing and health [5,6]. In adults, the experience of rejection seems to affect pain processing [7] and might be involved in the development of depression, borderline personality disorder, and anxiety disorders [8,9]. Previous studies have primarily investigated SP in adults, using the cyberball paradigm (which provides the most applied experimental induction possibilities for SP). Results have shown that there is a large overlap between the processing of physical and social pain and therefore, researchers have postulated a common pain network of physical and social pain [10].

## 2. Mental Imagery

Many mental health problems have their onset in adolescence [11]. To treat these disorders, it is important to understand the processes leading to such emotional dysfunctions. For adults, studies indicate that mental imagery (MI) plays a key role in the development and maintenance of psychological disorders [12]. These mental images not only occur visually but can appear in any sensory form like auditory, gustatory and olfactory, physical sensations, and tactile impressions [13] and might be associated with emotions like joy, sadness, anger, disgust, and fear (e.g., [12,14]). Mental images can contain parts of memories and maybe comparable to flashbacks (as in post-traumatic stress disorders, PTSD). But MI can also contain an image of a future event. These “flash-forwards” can trigger positive emotions (such as when one thinks with joy of a future event concerning a favorite hobby) or, as found in adult patients with depression, an image of one’s own suicide is linked to both distress *and* comfort [15,16]. Mental images are distressing and are linked to psychopathological symptoms when negative MI are frequent, associated with intense negative emotions and if they are experienced as intrusive and not controllable.

## 3. Mental Imagery in Psychopathology

There is also evidence that depression (in adolescents: [17,18,19,20,21]) and social anxiety disorder (SAD; adolescents: [22,23,24,25,26,27]) are linked to specific attributes of MI in adolescents (for a detailed review of the relevance of MI in psychopathology in children and adolescents, see [28]). 

Adolescents with depression were more likely to perceive negative mental images as more vivid and more often from an observer perspective, than adolescents without depression [17,19,20,21]. Concerning SAD, they discovered that negative self-images were a result, rather than a significant causal factor of SAD, and therefore may play a different role in the disorder among adolescents. Furthermore, neither valence nor content of self-images, but simply *having* self-images was a substantial factor in SAD during adolescence [22]. This finding contrasts with studies with adults, indicating that self-imagery does indeed play a causal role in SAD [29]. Further studies investigating MI in adolescents with SAD or social anxiety symptoms showed more frequent and more vivid self-images [25], that these images were associated with greater distress and that negative self-imagery was more often perceived with an observer perspective in patients with SAD [26]. These results are in accordance with [23,24]. In both studies, adolescents with high anxiety levels had more images in an observer perspective (experience the image from a third-person-perspective) than adolescents with low anxiety levels. The observer perspective might be part of a cognitive avoidance tendency, as in adult patients with PTSD as the observer perspective is linked to decreased emotions and might be less anxiety-provoking than a field perspective (experience the image in a first-person perspective) and therefore, might be an important maintaining factor [30].

## 4. Social Pain and Mental Imagery 

As there is an overlapping of the construct of social anxiety and SP as well as depression *and* SP, SP might also be associated with different characteristics of MI. Accordingly, patients suffering from SAD reported a fear of rejection and recovered more slowly from a current social rejection than patients without a SAD. Sensitivity to SP might also mediate the development of SAD [31,32]. Accordingly, sensitivity to social pain could be moderated by social anxiety symptoms as socially anxious individuals tend to perceive themselves and their social experiences more negatively [33].

The constructs of depression and SP are associated with one another since patients with depression also report a higher sensitivity to social rejection. The higher sensitivity to social rejection may lead to a key symptom of depression, namely social withdrawal. Girls reported a stronger internalizing reaction, such as withdrawal, as a response to social rejection than boys, whereas boys tended to display stronger externalizing reactions and expressed more anger and aggression [34,35].

Thus, the present pilot study aimed to explore the association between characteristics of MI and SP scores, as well as to determine the content of MI in adolescents with high SP scores. 

## 5. Hypotheses

Based on the literature search relating to adults and adolescents, we first hypothesized in negative images that higher SP-scores are linked to a higher frequency and vividness of negative images, as well as higher levels of reported emotions and distress. We also postulated that adolescents with a high-SP-sensitivity report images with social themes more often. 

Concerning positive images, we assumed that higher SP scores would be significantly associated with a lower frequency and vividness of positive images, as well as lower levels of associated joy. Concerning the use of perspective, we hypothesized that adolescents in a group with high SP scores would report more frequent use of observer perspective. Given that so far, no study has investigated the controllability of MI, we exploratively investigated whether or not the experienced controllability is linked to SP scores. Furthermore, we explored whether the vividness and several specific characteristics of MI would predict SP-sensitivity over and above the predictive power of depression and social anxiety. 

## 6. Method

We present data from an anonymous web-based study examining *N* = 80 adolescents, aged 14 to 20 years. The assessment was conducted using unipark (QuestBack GmbH, Berlin, Germany), an online survey software program. Participants were asked to generate two self-selected spontaneous images, a positive and a negative one. Questions were asked about the sensory qualities (visual, auditory, gustatory, and olfactory properties, physical sensations, and tactile impressions), the intensity of emotions (joy, fear, anger, sadness, disgust, shame, guilt), as well as the perspective of the two images. Additionally, the participants were requested to complete questionnaires about their experience of SP, depressive symptoms, and social anxiety symptoms. Participants specified whether or not their parents had agreed to the participation and data without parental consent was deleted. Permission from the ethics committee was obtained prior to the start of the study. 

## 7. Procedure and Recruitment 

The study was approved by the local ethics committee. Before starting the online survey, pilot data were collected for four adolescents (14 to 20 years old) to test the comprehensibility of the MI-questionnaire and the SP-questionnaire, neither of which had yet been used in an adolescent group. The time needed to complete the questionnaire was also stipulated. Participants were recruited using social media websites, e-mail distribution lists, online forums of soccer clubs, and local bulletin boards in Frankfurt, Heidelberg, and Ulm (Germany). In addition, flyers were distributed containing the goals of the study, as well as a QR-Code and hyperlink to the home page of the online survey. The survey started with a description of the study goals and participants were requested to provide informed consent. Participants specified whether or not their parents had agreed to the participation. Adolescents without parental consent were not allowed to complete the survey. Socio-demographic variables including age, gender, type of school, grade level, mother tongue, and existing mental diagnoses were requested. The online survey was conducted between January 2019 and July 2019. To increase motivation for participation, an iPad and cinema tickets were raffled. The email addresses (to contact the winners) of this raffle were saved completely independently of the questionnaire responses, to ensure anonymity.

## 8. Participants 

The first page of the online assessment was opened *n* = 436 times. Informed consent was provided by *n* = 149, and of the starters, *n* = 80 (54%) participants completed the full survey. Participants were excluded if they had indicated that they were visiting a special school or were currently experiencing an acute suicidal crisis. The required time for completing the assessments varied from 35 to 60 min. The mean age of patients was *M* = 17.66 years (*SD* = 1.76; range: 14–20; 75.3% girls, *n* = 61). Forty-six adolescents stated attending a high school, 26 declared attending university, seven participants were undertaking some form of training, and one adolescent was already working. Furthermore, 18 adolescents stated to have an immigration background. 

## 9. Measures

Mental Imagery Questionnaire for Youths (MIQ-Y, adapted by [36]; based on [37]). The MIQ-Y general structure was based on the items used in the *Imagery Interview* from [37]. In many studies with adults, the Imagery Interview has been shown to be a valid measure to assess mental imagery (e.g., [38]). As there are no validated tools for adolescents, we modified the *Imagery Interview*. A validation of the instrument could not be done due to the small sample size. 

A general description of a mental image was provided (e.g., Mental images are pictures which we can see with our “inner eye” like a photo or a film in our heads. They are sometimes based on real memories. The contents of these images can also seem very funny to you and do not necessarily have to be real, they can also be more like fantasy images. These mental images can refer to past events, the present, or the future. They can be pleasant or uncomfortable. The images sometimes appear although you do not want them to. Many people have such inner mental images some have them often, some seldom. Afterward, participants were asked to imagine and describe two self-selected spontaneous mental images, first a positive one and then a negative one. After each specific image, they described what they experienced in each sensory impression (including visual, auditory, gustatory, and olfactory properties, physical, and tactile sensations) and rated the clarity on a five-step scale (from 1 = no image at all to 5 = as vivid as real life) and also the strength of the associated emotions (joy, anger, fear, sadness, disgust, shame, guilt), controllability (the control over the mental image itself) on a scale from 0 (no associated emotions, no distress, no control) to 10 (very strong emotions, high distress, full control). Respondents are also asked for the perspective (field, observer, combination of field and observer, not specified). Furthermore, participants report the frequency of occurrence in the last six months (from 1 = almost never, only if I’m asked to think about it to 5 = always, at least once a day).

The different descriptions of the participants were divided into categories according to [39], based on an inductive procedure. The two superordinate categories for positive images are social reward and physical reward. For negative images, the two superordinate categories are physical threat and interpersonal threat. Five categories for positive images (social reward: *enjoying time with family or friends, romantic situations*; physical reward: *enjoying nature, pursuing a sport*) and seven categories for negative images (interpersonal threat: *conflicts with friends/family, death of a close relative, loneliness, failure in a performance situation, social embarrassment*; physical threat: *unintended/accidental physical injury to oneself; physical harm of another person*) were identified. 

Social Pain Questionnaire (SPQ; [40]). The *Social Pain Questionnaire* consists of 18 items, each involving a statement about the perception of SP (e.g., if I am excluded by a group, it offends me a lot; if someone ignores me, it hurts me a lot). Participants rate these statements on a 5-point scale (ranging from *not applicable* to *fully applicable*). Internal consistency of the questionnaire has been established as high (Cronbach’s *α* = 0.95). Past research has also confirmed highly significant, positive correlations and good convergent validity between the sensitivity to SP and depression (*r =* 0.55, *p* < 0.01), social anxiety (*r =* 0.64, *p* < 0.01), and an anxious (*r* = 0.34, *p* < 0.01) and avoiding attachment style (*r =* 0.49, *p* < 0.01) in an adult-sample (*M* = 32.61 years, *SD* = 11.13). This questionnaire has not yet been validated for youths [40]. The contents of the questionnaire were adjusted (changing words from “valid” to “important”, “colleagues” to “classmates”, providing examples of somatic syndromes like headache or nausea) to ensure age-appropriateness. Based on a factor analysis, we were able to identify that in our sample, the SPQ is based on a one-dimensional construct, and the internal consistency of the total scale in our sample is high (Cronbach’s *α =* 0.938).

Beck Depression Inventory (BDI-II; [41]). The BDI-II assesses depressive symptoms such as changes in appetite, sleeping problems, and bad moods. Twenty-one items have to be rated on a four-point scale. The BDI-II is a reliable instrument for assessing depressive symptoms in clinical and non-clinical populations (from 13 years old). The BDI-II was found to have a Cronbach’s *α* ≥ 0.84 for non-depressed and acutely depressed samples, as well as adequate content validity and sufficient retest reliability in non-clinical samples [42].

Social Phobia Inventory (SPIN); originally developed by [43]; German version by [44]. The German version of the SPIN consists of 17 items that assess social anxiety symptoms. Participants rate these items on a 5-point Likert scale ranging from 1 (not stressful) to 5 (extremely stressful). The measure displays high internal consistency (Cronbach’s *α* = 0.89 and *α =* 0.95) and good retest reliability *r_tt_* = 0.85 in populations aged 14 to 93 years [44]. 

## 10. Statistical Analyses

Prior to the data analysis, participants were screened based on previously outlined exclusion criteria (no participant had to be excluded), and the data set was checked for completeness and correctness, as well as the fit between the distributions and the assumptions of multivariate analysis, according to [45]. Because all of the responses were ‘forced-choice’, no missing data was generated. We also checked and can confirm that our data meet the requirements (homogeneous variability and normal distributions) for the parametric analysis.

To examine the degree of association between SP scores and MI characteristics, we used correlation analyses. The following variables were included in the correlation analysis: imagery frequency, imagery vividness, controllability, emotional distress, as well as the strength of associated emotions (joy, fear, anger, sadness, disgust, shame, guilt). We conducted two correlation analyses, one for positive imagery and one for negative. To explore the influence of the covariates *social anxiety-* and *depression scores,* as well as *gender,* we used partial correlation analysis. For each analysis, we included all individuals (*n* = 80).

To compare students with different levels of SP concerning the occurrence of MI and the use of perspective in positive and negative imagery, the sample was split into quartiles, in line with [46]. The *high symptom* group (high-SP group) was based on the top 25% of the scores in the social pain measure (SPQ) and the *low symptom* group (low-SP group) consisted of the participants scoring in the lowest 25% on the measure. The limit for the lowest 25% was a sum of 39 points, and for the highest 25%, the sum was 61. After splitting the sample into quartiles, the high- and low-symptom groups each consisted of 20 individuals. The groups were compared demographic variables such as age and sex, using an independent sample *t*-test and *Fisher–Yates* tests. To examine differences in *imagery perspective* (options: observer-, field perspective, the combination of both perspectives, not specified) and *occurrence* between the high-SP group and low-SP group, we used *χ*^2^-tests for positive and negative MI. To investigate the differences in the content of negative and positive MI between SP groups, *χ*^2^-test and *Fisher–Yates* tests were conducted. With a coefficient of determination of *R²* = 0.18, statistical power of 0.9, α = 0.05, a sample size of *n* = 76 would be needed for a significant overall model for the comparison of contents between SP-groups.

Two sequential linear regression analyses, one for negative MI and one for positive MI, were computed to examine whether SP-, depression- or social anxiety scores significantly predict MI characteristics (frequency, vividness, controllability, associated emotions). 

## 11. Results

### 11.1. Correlation Analysis of Characteristics of Negative Imagery and Social Pain-Scores 

To explore the link between SP-scores and the variables imagery frequency, imagery vividness, controllability, emotional distress and emotions (joy, fear, anger, sadness, disgust, shame, guilt) in negative imagery, a correlation analysis was conducted. Even after including social anxiety- and depression-scores in a partial analysis, significant correlations were found. As significant differences between high- and low-SP groups appeared, *gender* was also included as a covariate. Table 1 displays significant correlations of every partial analysis. 

### 11.2. Correlation Analysis of Characteristics of Positive Imagery and Social Pain-Scores 

To explore the link between SP-scores and the variables imagery frequency, imagery vividness, controllability, emotional distress, and *emotions* (joy, fear, anger, sadness, disgust, shame, guilt) in positive imagery, a correlation analysis was conducted. No significant correlations (*p* ≥ 0.05) were found for SP scores and characteristics of positive images. 

### 11.3. Prediction of Characteristics of Positive and Negative MI by Clinical Measures

Using all individuals, (*n* = 80), a sequential linear regression analysis was used to examine whether SP-, depression-, or social anxiety-scores predict negative MI characteristics in the most suitable manner. One more sequential analysis was computed to explore whether SP-, depression-, or social anxiety scores predict positive MI characteristics.

In negative images, SP scores made the largest contribution to the prediction of the *imagery vividness* (*β* = 0.31, *p* = 0.006), *emotional distress* (*β* = 0.34, *p* = 0.002) as well as *anxiety* (*β* = 0.37, *p* = 0.001), *shame* (*β* = 0.34, *p* = 0.002), *sadness* (*β* = 0.29, *p* = 0.01), and *anger* (*β* = 0.23, *p* = 0.04). *Imagery frequency* (*β* = 0.43, *p* ≤ 0.001) was most accurately predicted by social anxiety-scores. Feelings of *guilt* were also most accurately predicted by SP-scores (*β* = 0.43, *p* ≤ 0.001) and depression-scores (*β* = 0.25, *p* = 0.02). For *controllability*, *joy*, and *disgust*, no clinical measure was found to predict these variables reliably.

For positive images, *emotional distress* (*β* = 0.26, *p* = 0.02), *controllability* (*β* = −0.33, *p* = 0.003), and *joy* (*β* = −0.24, *p* = 0.03), were most accurately predicted by depression-scores. For *imagery vividness* and *frequency*, *anxiety*, *disgust*, and *shame*, no clinical measure was found to predict these variables accurately. 

### 11.4. Group Differences among SP-Groups

To compare students with different levels of SP concerning the occurrence of positive and negative imagery and the use of perspective, the sample was split into quartiles. A *Fisher–Yates* test showed a significant difference in *gender* (*p* = 0.01) and *education* (*p* = 0.02) between the SP-groups. The high-SP group includes more females and fewer high school students than in the low-SP group. No significant differences in *age* emerged between the groups (*p* = 0.74). Individuals in the high-SP group show not only significantly higher scores for the SP questionnaire, but also for the depression (*p* ≤ 0.001) and social anxiety questionnaires (*p* ≤ 0.001). Table 2 shows the demographic characteristics and clinical measures of the two groups.

Differences in occurrence and content of MI between high- and low-SP groups. The results showed significant differences in the occurrence of negative mental images between the high- and low-SP groups (*χ*^2^_(1,39)_ = 5.63 *p* = 0.02). Of 20 individuals in the high-SP group, 19 reported having negative images, in the low-SP group 13 adolescents reported experiencing negative images, seven adolescents in the low-SP group did not report negative imagery. 

Concerning the superordinate content of negative images, differences were significant in a *χ*^2^-test (*χ*^2^_(1,38)_ = 5.78 *p* = 0.029). Adolescents in the high-SP group were more likely to report images about *interpersonal threat*. A *Fisher–Yates* test investigating the difference in subcategories showed no significant group differences (*χ*^2^_(6,32)_ = 13.77; *p* = 0.055). Table 3 shows the frequency distribution of the different contents for negative images. 

Concerning the experience of positive images, a *χ*^2^-test showed no significant difference in the occurrence of positive mental images between the high- and low-SP groups (*χ*^2^_(1,39)_ = 1.026 *p* = 1.00). All 20 individuals in the low-SP group reported having positive images, in the high-SP group, 19 of the 20 adolescents reported experiencing positive images. 

Concerning the contents of positive images, there were no significant differences either in the *χ*^2^ -test (for superordinate categories: *χ*^2^_(1,38)_ = 0.1.48; *p* = 0.224) or in the Fisher–Yates tests (for subcategories: *χ*^2^_(4,39)_ = 3.69 *p* = 0.450) between the two SP-groups. Table 4 shows the frequency distribution of the different contents for positive images.

Differences in adopted imagery perspective between high- and low-SP groups. To examine differences between the high- and low-SP groups in the use of the perspective options (observer-, field perspective, the combination of both perspectives, not specified), we used a *Fisher–Yates* test. The test showed a significant difference in the use of perspective in negative images between the high- and low-SP groups (*χ*^2^_(3,39)_ = 10.41; *p* = 0.01). Table 5 shows the frequency distribution of the different perspective options. 

Most of the individuals in the low-SP-group reported perceiving the image in a field-perspective (50%), whereas one person reported perceiving it in a combination of both perspectives. Thirty percent could not specify in which perspective they perceived the image. In the high-SP group, adolescents reported observing the negative images in a field-perspective (40%) and a combination of both perspectives (45%). Differences in positive images were not found. 

## 12. Discussion

The study aimed to investigate the link between social pain (SP) and mental imagery (MI). This is the first study to investigate imagery characteristics and the sensitivity to SP. 

### 12.1. Social Pain and Negative Mental Imagery 

It emerged that higher SP scores in adolescents were associated with a significantly higher imagery frequency, imagery vividness, associated emotional distress, fear, anger, sadness, shame, and especially feelings of guilt. Furthermore, higher SP scores were associated with less control over the negative image. Statistical analysis showed moderate correlations between the variables. Even after including social anxiety-, depression scores, and gender as covariates in a partial analysis, the significant correlations were sustained between SP-scores and associated fear, sadness, and guilt. Especially guilt, as a social emotion was associated in this context. This suggests that individuals with a high sensitivity to SP might have not reachable and very high internal moral standards [47]. The adjustment of these extreme standards could be a suitable goal in psychotherapy with patients with high-SP sensitivity. The result that higher SP scores were associated with less control over the negative image was also persistent. Therefore, SP sensitivity might play an important role in explaining variances in these characteristics. Furthermore, most characteristics of negative imagery were predicted most accurately by SP scores. Only imagery frequency was most accurately predicted by social anxiety scores. Therefore, SP sensitivity seems to be an important factor when investigating and dealing with MI in youths. Using methods that aim to reduce SP-sensitivity (like behavioral experiments, cognitive methods) might also have a beneficial effect on MI. Alternatively, techniques in therapy focusing on changing negative MI (like imagery rescripting) might also help to reduce SP-sensitivity. Looking at the results concerning controllability, it might also be appropriate to consider techniques in therapy to increase controllability over negative imagery in patients with higher sensitivity to SP. Our results also support the assumption that adolescence is a fragile life period in which social exclusion hurt particularly. The link between MI and SP reveals the strong emotional impact of social exclusion in adolescents, endorsing the use of methods reducing SP and gaining control over MI in therapy. The implementation of training to improve social skills, as these training might help to reinterpret difficult and ambiguous social situations in a more functionally and less painfully way. 

### 12.2. Social Pain and Positive Mental Imagery 

For positive images, characteristics were most accurately predicted by depression scores. Higher depression scores were associated with less controllability and joy, and more distress in fact due to positive images. This is in accordance with previous research exploring depression and MI, as depression seems to affect positive imagery and not only on negative imagery [15,20,48]. Thus, positive MI characteristics were linked most of all to depressive symptoms. However, causal attributions cannot be made, as the results only reveal associations between MI and SP.

The results also indicate a significantly increased frequency of negative images in adolescents with high-SP-sensitivity. There were no significant differences between SP groups in the frequency of positive images. Interestingly, the content of negative images involved close others more frequently in the high-SP- than in the low-SP group. A negative or positive image of an unpleasant or pleasant social situation could therefore be particularly hurtful or enjoyable for people with a high sensitivity for SP compared to positive images without a social theme. This needs to be investigated in an experimental design.

### 12.3. Social Pain and Attachment Styles

Since different attachment styles appear to be associated with different levels of sensitivity to social pain [49] and since the present study shows a relationship between the characteristics of mental images and sensitivity to SP, attachment styles may also influence the appearance and characteristics of mental images. For example, an insecure attachment style is strongly related to a high sensitivity to SP and could thus also be associated with particularly distressing and/or more frequent negative images. This hypothesis also needs further investigation.

### 12.4. Social Pain and Perspective of Mental Imagery

The results also showed significant differences in the use of perspective in negative MI between adolescents with high- and low SP-sensitivity. Remarkably, there was a more frequent use of the combination of both perspectives in the high-SP group. This raises questions about previous research comparing only field and observer perspectives, but not the combination of both. In previous studies, patients with different disorders reported having either field- or observer perspectives (e.g., SAD: [23,24,26]; depression: [17]; PTSD: [50]). In our sample, the combination of both perspectives was associated with a higher level of psychopathological symptoms (as the high- and low-SP groups also differed significantly in depression- and social anxiety scores). Possibly, using the combination of both perspectives is already an indication of higher psychopathological symptoms in general. 

### 12.5. Social Pain and Physical Pain

As previous studies in adults showed a large overlap in the processing of physical and social pain, it could be postulated that a stimulus might have a parallel effect on the sensitivity of one type of pain and the other [51]. Thus, the influence of social support on physical pain is conceivable, as is the influence of painkillers on SP and consequently on MI. For example, the intake of paracetamol was shown to have an inhibitory effect on the perceived SP after social rejection and reduced anterior cingulate cortex activity [52]. Treating negative MI could therefore also lead to a reduction of SP and physical pain which might support the use of helpful imaginations during physical procedures. 

## 13. Limitations

The study has several limitations. As already mentioned, nothing can be said about the causality of the relationship between SP sensitivity and characteristics of mental images, as we used a cross-sectional design in this study. Given that we recruited an analog adolescent sample and there were no external clinical assessments and no detailed diagnostic face-to-face interviews (and therefore no diagnosis can be made), there is only limited applicability and generalizability of any conclusions drawn, to clinical samples. As we only used self-report measures there is a lack of verifiability of the objectivity. Because of its exploratory nature, this study could be a preliminary study to an experimental study where one could manipulate social exclusion (e.g., via the cyberball) and evaluate the effects on mental imagery.

Although we gathered pilot data and tested the comprehensibility and clarity of the questionnaires, we cannot ignore the fact that the questionnaires may have been understood differently in terms of language (e.g., unsuitable wording, [53]), lack of adequate reading ability [54], or native language [55] as this could be common in adolescents compared to adults. The use of questionnaires (and not face-to-face interviews) might not be suitable for measuring such a complicated and nonfigurative construct as MI. Youths might have more problems reporting emotions, cognitions, and other mental processes, compared to adults, as they might have a less pronounced self-awareness. As most participants dropped out after the MiQ-Y, the questions may have been too conceptual for some of the adolescents, explaining the high educational status of our final sample. The responses of the participants in this study, who completed the whole survey, did not indicate that they had trouble understanding any questions, although the MIQ-Y was developed based on a semi-structured interview (Hackmann et al., 2000) and not based on a questionnaire. Although the interview of [37] has been widely used in adults and has shown to be a valid instrument, the validity of our measure is questionable. 

Furthermore, the sample size is rather small, especially for the comparison of the contents of MI, which especially limits the informative value of the results concerning contents. Furthermore, our sample has a relatively high social status and education, as more than half are attending advanced schools or universities. Additionally, there are several limitations because the data are from an online survey. Certain age and social groups as well as individuals are more likely to take part in online surveys. For example, participants with a lower level of education might not respond to such a survey, which might also explain the generally high educational status [56]. We also cannot ensure that the information given on age or gender, for example, is true. To guarantee that a participant only completed the survey only one time, every IP-address only could participate once. However, we still cannot definitely ensure that participants completed the survey a second time. Furthermore, we could not control current mood or any kind of environmental emotional priming effects such as actual experience of social pain (e.g., bullying). 

## 14. Conclusions

Despite these limitations, the current investigation is among the first to examine MI and its link to SP. Higher scores of SP were significantly associated with a higher score for anxiety, sadness, and guilt and with less control over negative MI. SP-scores seem to predict differences in negative MI more appropriately than social anxiety- or depression scores indicating that differences in SP-sensitivity need to be controlled in studies investigating methods using MI for treatment of mental disorders as possible group differences may falsify outcome measures. The relationship between SP-sensitivity and MI characteristics may explain why negative mental imagery manifests in childhood and adolescence and may contribute to the development of a mental disorder. Furthermore, the link between MI and SP reveals the strong emotional impact of social exclusion in youths. Adolescents in the high-SP group reported using the combination of both perspectives in negative images more often. Using the combination of both perspectives in MI might indicate more severe psychopathological symptoms in general. As this is the first study dealing with the combination of perspectives, the results raise questions about previous results comparing each perspective individually. Consequently, replications with clinical adolescent samples are needed, as well as longitudinal assessments of the MI, to deal satisfactorily with causal issues. The present pilot study also constitutes a starting point for future investigations of the association between SP and characteristics of MI. 

## Figures and Tables

**Table 1:** Significant correlations between SP-scores and characteristics of negative MI.

	Correlations with SPQ-Scores		SPIN-Scores Partialled Out		BDI-Scores Partialled Out		Gender Partialled Out	
	*r*	*p*	*r*	*p*	*r*	*p*	*r*	*p*
Frequency	0.367	0.001			0.268	0.017	0.312	0.005
Vividness	0.307	0.006					0.231	0.040
**Controllability**	**−0.379**	0.001 **	**−0.253**	**0.024**	**−0.313**	**0.005**	**−0.325**	**0.003**
Emotional Distress	0.344	0.002			0.261	0.020	0.284	0.011
Emotions								
**fear**	**0.365**	**0.001**	**0.304**	**0.006**	**0.315**	**0.005**	**0.315**	**0.005**
**guilt**	**0.433**	0.001 **	**0.331**	**0.003**	**0.327**	**0.003**	**0.434**	0.001 **
shame	0.337	0.002			0.322	0.004	0.294	0.008
**sadness**	**0.292**	**0.009**	**0.224**	**0.047**	**0.224**	**0.048**	**0.241**	**0.032**
anger	0.229	0.041						

Note. SP = Social Pain; MI = Mental imagery; SPQ = Social-Pain-Questionnaire. BDI-II = Becks-Depression-Inventory. SPIN = Social Phobia Inventory. Correlations that sustained are in bold. ** p ≤ 0.001.

**Table 2:** Demographic characteristics and clinical measures.

	Low-SP *n* (%) or Mean (*SD*)	High-SP *n* (%) or Mean (*SD*)	Statistic
Age	17.50 (1.91)	17.50 (1.81)	*t*_(38)_ = −0.34; *p* = 0.74
Gender (%)	10 Female (50%) 9 Male (45%) 1 not specified (5%)	18 (90%) 1 Male (5%) 1 not specified (5%)	*χ*^2^_(2, 38)_ = 8.99; *p* = 0.01 *
Education			
Basic School	1 (5%)	3 (15%)	
Advanced School	15 (75%)	7 (35%)	
University	2 (10%)	9 (45%)	
Training	1 (5%)	1 (5%)	
Working	1 (5%)	0	*χ*^2^_(2, 38)_ = 9.35; *p* = 0.02 *
Clinical measures			
SPQ	32.10 (5.05)	70.10 (7.57)	*t*_(38)_ = −18.69; *p* ≤ 0.001 **
BDI-II	7.70 (6.95)	18.50 (10.42)	*t*_(38)_ = −3.86; *p* ≤ 0.001 **
SPIN	9.55 (6.84)	23.70 (13.21)	*t*_(38)_ = −4.25; *p* ≤ 0.001 **

Note. SP = Social Pain; Low-SP = Group of adolescents with a low score on a social pain measure; High-SP = Group of adolescents with a high score on a social pain measure. SPQ = Social-Pain-Questionnaire. BDI-II = Becks-Depression-Inventory. SPIN = Social Phobia Inventory. ** p ≤ 0.001. * *p* < 0.05.

**Table 3:** Frequency distribution of contents in negative mental imagery for social pain.

Content of Negative Images	Low-SP *n* (%)	High-SP *n* (%)
Interpersonal threat	5 (25%)	14 (70%)
Conflicts with friends/family (e.g., “crying at my desk, reading an awful message from my boyfriend”)	1	3
Loneliness (e.g., “being alone, not being able to speak”)	1	2
Social embarrassment (e.g., “pupils making fun of me, insulting me, laughing at me)	1	6
Failure in a performance situation (e.g., “sitting in my final exams, forgot everything I’ve learned”)	2	3
Physical threat	9 (45%)	4 (20%)
Physical harm to another person (e.g., “a friend of mine falls down the stairs at school, hearing his crying and falling sounds”)	1	3
Death of someone I care about (e.g., “seeing my grandpa lying in a coffin”)	5	1
Accidental/unintended physical injury of oneself (e.g., “getting a door slammed into my face, feel blood running down my face”)	3	0
No image	6 (30%)	2 (10%)

Note. Content of negative mental images in an adolescent sample aged 14–20 years. SP = Social Pain; Low-SP = Group of individuals with a low score on social pain measure; High-SP = Group of individuals with a high score on a social pain measure.

**Table 4:** Frequency distribution of contents in positive mental imagery for social pain.

Content of Positive Images	Low-SP *n* (%)	High-SP *n* (%)
Social reward	10 (50%)	14 (70%)
Enjoying time with family or friends (e.g., “it’s Christmas eve and I’m playing ball with my grandpa”)	7	11
Romantic situation (e.g., “kissing my girlfriend, smelling her perfume and feeling her lips”)	3	3
Physical reward	10 (50%)	5 (25%)
Enjoying nature (e.g., “walking through the forest in good weather and enjoying the fresh air”)	7	3
Pursuing a sport (e.g., “being on a horse farm and taking care of my favorite pony”)	3	2
No image	0 (0%)	1 (5%)

Note. Content of positive mental images in an adolescent sample aged 14–20 years. Note: SP = Social Pain; Low-SP = Group of individuals with a low score on the social pain measure; High-SP = Group of individuals with a high score on the social pain measure.

**Table 5:** Frequency distribution of perspective options in negative mental imagery for social pain.

Perspective Options	Low-SP *n* (%)	High-SP *n* (%)
Field perspective	10 (50%)	8 (40%)
Observer Perspective	3 (15%)	2 (10%)
Combination of both	1 (5%)	9 (45%)
Not specified	6 (30%)	1 (5%)

Note: SP = Social Pain; Low-SP = Group of individuals with a low score on the social pain measure; High-SP = Group of individuals with a high score on a social pain measure.

## Data Availability

The data presented in this study are available on request from the corresponding author. The data are not publicly available due to ethical reasons.

## References

[1] Blakemore S.-J., Choudhury S. (2006). Development of the adolescent brain: Implications for executive function and social cognition. J. Child Psychol. Psychiatry.

[2] Sebastian C.L., Tan G.C., Roiser J.P., Viding E., Dumontheil I., Blakemore S.-J. (2011). Developmental influences on the neural bases of responses to social rejection: Implications of social neuroscience for education. NeuroImage.

[3] Dalgleish T., Walsh N.D., Mobbs D., Schweizer S., van Harmelen A.-L., Dunn B., Dunn V., Goodyer I., Stretton J. (2017). Social pain and social gain in the adolescent brain: A common neural circuitry underlying both positive and negative social evaluation. Sci. Rep..

[4] Leary M.R., Forgas J.P. (2000). Affect, cognition, and the social emotions. Feeling and Thinking: The Role of Affect in Social Cognition.

[5] Baumeister R.F., Leary M.R. (1995). The need to belong: Desire for interpersonal attachments as a fundamental human motivation. Psychol. Bull..

[6] Williams K.D. (2007). Ostracism. Annu. Rev. Psychol..

[7] Bernstein M.J., Claypool H.M. (2011). Social Exclusion and Pain Sensitivity. Pers. Soc. Psychol. Bull..

[8] Hawthorne G. (2007). Perceived social isolation in a community sample: Its prevalence and correlates with aspects of peoples’ lives. Soc. Psychiatry Psychiatr. Epidemiol..

[9] Stanley B., Siever L.J. (2010). The Interpersonal Dimension of Borderline Personality Disorder: Toward a Neuropeptide Model. Am. J. Psychiatry.

[10] Williams K.D., Cheung C.K.T., Choi W. (2000). Cyberostracism: Effects of being ignored over the Internet. J. Pers. Soc. Psychol..

[11] Paus T., Keshavan M., Giedd J.N. (2008). Why do many psychiatric disorders emerge during adolescence?. Nat. Rev. Neurosci..

[12] Holmes E.A., Mathews A. (2010). Mental imagery in emotion and emotional disorders. Clin. Psychol. Rev..

[13] Andrade J., May J., Deeprose C., Baugh S.-J., Ganis G. (2013). Assessing vividness of mental imagery: The Plymouth Sensory Imagery Questionnaire. Br. J. Psychol..

[14] Blackwell S.E. (2019). Mental imagery: From basic research to clinical practice. J. Psychother. Integr..

[15] Holmes E.A., Crane C., Fennell M.J., Williams J.M.G. (2007). Imagery about suicide in depression—“Flash-forwards”?. J. Behav. Ther. Exp. Psychiatry.

[16] Moritz S., Hörmann C.C., Schröder J., Berger T., Jacob G.A., Meyer B., Holmes E.A., Späth C., Hautzinger M., Lutz W. (2013). Beyond words: Sensory properties of depressive thoughts. Cogn. Emot..

[17] Kuyken W., Howell R. (2006). Facets of autobiographical memory in adolescents with major depressive disorder and never-depressed controls. Cogn. Emot..

[18] McKinnon A.C., Nixon R.D., Brewer N. (2008). The influence of data-driven processing on perceptions of memory quality and intrusive symptoms in children following traumatic events. Behav. Res. Ther..

[19] Meiser-Stedman R., Dalgleish T., Yule W., Smith P. (2012). Intrusive memories and depression following recent non-traumatic negative life events in adolescents. J. Affect. Disord..

[20] Pile V., Lau J.Y. (2018). Looking forward to the future: Impoverished vividness for positive prospective events characterises low mood in adolescence. J. Affect. Disord..

[21] Pile V., Lau J.Y. (2020). Intrusive images of a distressing future: Links between prospective mental imagery, generalized anxiety and a tendency to suppress emotional experience in youth. Behav. Res. Ther..

[22] Alfano C.A., Beidel D.C., Turner S.M. (2008). Negative Self-Imagery Among Adolescents with Social Phobia: A Test of an Adult Model of the Disorder. J. Clin. Child Adolesc. Psychol..

[23] Hignett E., Cartwright-Hatton S. (2008). Observer Perspective in Adolescence: The Relationship with Social Anxiety and Age. Behav. Cogn. Psychother..

[24] Ranta K., Tuomisto M.T., Kaltiala-Heino R., Rantanen P., Marttunen M. (2013). Cognition, Imagery and Coping among Adolescents with Social Anxiety and Phobia: Testing the Clark and Wells Model in the Population. Clin. Psychol. Psychother..

[25] Schreiber F., Höfling V., Stangier U., Bohn C., Steil R. (2012). A Cognitive Model of Social Phobia: Applicability in a Large Adolescent Sample. Int. J. Cogn. Ther..

[26] Schreiber F., Steil R. (2012). Haunting self-images? The role of negative self-images in adolescent social anxiety disorder. J. Behav. Ther. Exp. Psychiatry.

[27] Vassilopoulos S.P., Moberly N.J., Douratsou K.-M. (2011). Social Anxiety and the Interaction of Imagery and Interpretations in Children: An Experimental Test of the Combined Cognitive Biases Hypothesis. Cogn. Ther. Res..

[28] Schwarz S., Grasmann D., Schreiber F., Stangier U. (2020). Mental Imagery and its Relevance for Psychopathology and Psychological Treatment in Children and Adolescents: A Systematic Review. Int. J. Cogn. Ther..

[29] Hirsch C.R., Mathews A., Clark D.M., Williams R., Morrison J.A. (2006). The causal role of negative imagery in social anxiety: A test in confident public speakers. J. Behav. Ther. Exp. Psychiatry.

[30] Kenny L.M., Bryant R.A. (2007). Keeping memories at an arm’s length: Vantage point of trauma memories. Behav. Res. Ther..

[31] Fung K., Alden L.E. (2017). Once Hurt, Twice Shy: Social Pain Contributes to Social Anxiety. Emotion.

[32] Zadro L., Boland C., Richardson R. (2006). How long does it last? The persistence of the effects of ostracism in the socially anxious. J. Exp. Soc. Psychol..

[33] Moscovitch D.A., Orr E., Rowa K., Reimer S.G., Antony M.M. (2009). In the absence of rose-colored glasses: Ratings of self-attributes and their differential certainty and importance across multiple dimensions in social phobia. Behav. Res. Ther..

[34] DeRosier M.E., Kupersmidt J.B., Patterson C.J. (1994). Children’s Academic and Behavioral Adjustment as a Function of the Chronicity and Proximity of Peer Rejection. Child Dev..

[35] London B., Downey G., Bonica C., Paltin I. (2007). Social Causes and Consequences of Rejection Sensitivity. J. Res. Adolesc..

[36] Schwarz S., Schreiber F. (2016). Fragebogen zur Erfassung mentaler Bilder bei Jugendlichen (FEMB-J).

[37] Hackmann A., Clark D., McManus F. (2000). Recurrent images and early memories in social phobia. Behav. Res. Ther..

[38] Day S., Holmes E., Hackmann A. (2004). Occurrence of imagery and its link with early memories in agoraphobia. Memory.

[39] Mayring P. (2002). Einführung in Die Qualitative Sozialforschung.

[40] Stangier U., Schüller J., Brähler E. (2021). Development and validation of a new instrument to measure social pain. Sci. Rep..

[41] Beck A.T., Steer R.A., Brown G.K. (1992). Manual for the Beck Depression Inventory-II.

[42] Kühner C., Bürger C., Keller F., Hautzinger M. (2007). Reliabilität und Validität des revidierten Beck-Depressionsinventars (BDI-II). Der Nervenarzt.

[43] Connor K.M., Davidson J.R.T., Churchill E., Sherwood A., Foa E., Weisler R.H. (2000). Psychometric properties of the Social Phobia Inventory (SPIN). Br. J. Psychiatry.

[44] Consbruch K., Stangier U., Heidenreich T. (2016). SOZAS: Skalen zur Sozialen Angststörung: Soziale-Phobie-Inventar (SPIN), Soziale-Interaktions-Angst-Skala (SIAS), Soziale-Phobie-Skala (SPS), Liebowitz-Soziale-Angst-Skala (LSAS): Manual, 1. Auflage.

[45] Tabachnick B.G., Fidell L.S. (2007). Using Multivariate Statistics.

[46] Hodson K.J., McManus F.V., Clark D.M., Doll H. (2008). Can Clark and Wells’ (1995) cognitive model of social phobia be applied to young people. Behav. Cogn. Psychother..

[47] Ferguson T.J., Stegge H., Miller E.R., Olsen M.E. (1999). Guilt, shame, and symptoms in children. Dev. Psychol..

[48] Morina N., Deeprose C., Pusowski C., Schmid M., Holmes E.A. (2011). Prospective mental imagery in patients with major depressive disorder or anxiety disorders. J. Anxiety Disord..

[49] Chester D.S., Pond R.S.J., Richman S.B., DeWall C.N. (2012). The optimal calibration hypothesis: How life history modulates the brain’s social pain network. Front. Evol. Neurosci..

[50] McIsaac H.K., Eich E. (2004). Vantage Point in Traumatic Memory. Psychol. Sci..

[51] Macdonald G., Leary M.R. (2005). Why Does Social Exclusion Hurt? The Relationship Between Social and Physical Pain. Psychol. Bull..

[52] DeWall C.N., MacDonald G., Webster G.D., Masten C.L., Baumeister R.F., Powell C., Combs D., Schurtz D.R., Stillman T.F., Tice D.M. (2010). Acetaminophen Reduces Social Pain. Psychol. Sci..

[53] Campbell M.A., Rapee R.M. (1996). Current Issues in the Assessment of Anxiety in Children and Adolescents: A Developmental Perspective. Behav. Chang..

[54] Vasey M.W., Dalgleish T., Silverman W.K. (2003). Research on information-processing factors in child and adolescent psychopathology: A critical commentary. J. Clin. Child Adolesc. Psychol..

[55] Alfano C., Beidel D.C., Turner S.M. (2002). Cognition in childhood anxiety: Conceptual, methodological, and developmental issues. Clin. Psychol. Rev..

[56] Blasius J., Brandt M. (2009). Repräsentativität in online-befragungen. Umfrageforschung.

